# Stress hyperglycemia ratio as an important predictive indicator for severe disturbance of consciousness and all-cause mortality in critically ill patients with cerebral infarction: a retrospective study using the MIMIC-IV database

**DOI:** 10.1186/s40001-025-02309-9

**Published:** 2025-01-27

**Authors:** Xiaosheng Li, Li Guo, Yuzhen Zhou, Churan Yuan, Yong Yin

**Affiliations:** Department of Rehabilitation Medicine, The Affiliated Hospital of Yunnan University, No. 176 Qingnian Road, Wuhua District, Kunming, Yunnan China

**Keywords:** Stress hyperglycemia ratio (SHR), Severe disturbance of consciousness, All-cause mortality, Stroke, MIMIC-IV

## Abstract

**Background:**

Stress hyperglycemia ratio (SHR) has been linked to prognosis of cerebrovascular diseases. Nevertheless, the association between SHR and severe disturbance of consciousness (DC) and mortality among patients with cerebral infarction remains explored. This study seeks to assess the predictive potential of SHR for severe DC and mortality among patients with cerebral infarction.

**Methods:**

We identified individuals diagnosed with cerebral infarction within the MIMIC-IV database. We employed logistic regression to examine the correlation between the SHR index and the severity of patients' consciousness disturbance, as well as in-hospital mortality. Furthermore, we employed restricted cubic spline curves to explore potential non-linear relationships between the SHR index and outcome measures. To assess the predictive performance of the SHR index and admission blood sugar level on outcome indicators, we compared receiver operating characteristic (ROC) curves.

**Results:**

A non-linear relationship existed between SHR and the risk of severe disturbance of consciousness, while there was a linear relationship with all-cause mortality. The AUC value for predicting severe disturbance of consciousness by the SHR index is 0.5419 (95% CI: 0.5188–0.5661). The AUC value for predicting in-hospital mortality based on the SHR index is 0.6264 (95% CI: 0.5881–0.6662). It is superior to single admission blood sugar level. In addition, SHR has an incremental impact on evaluating various diseases in predicting severe disturbance of consciousness and all-cause mortality in critically ill patients with cerebral infarction.

**Conclusions:**

SHR is an important predictive indicator for severe disturbance of consciousness and all-cause mortality of patients with cerebral infarction.

**Graphical Abstract:**

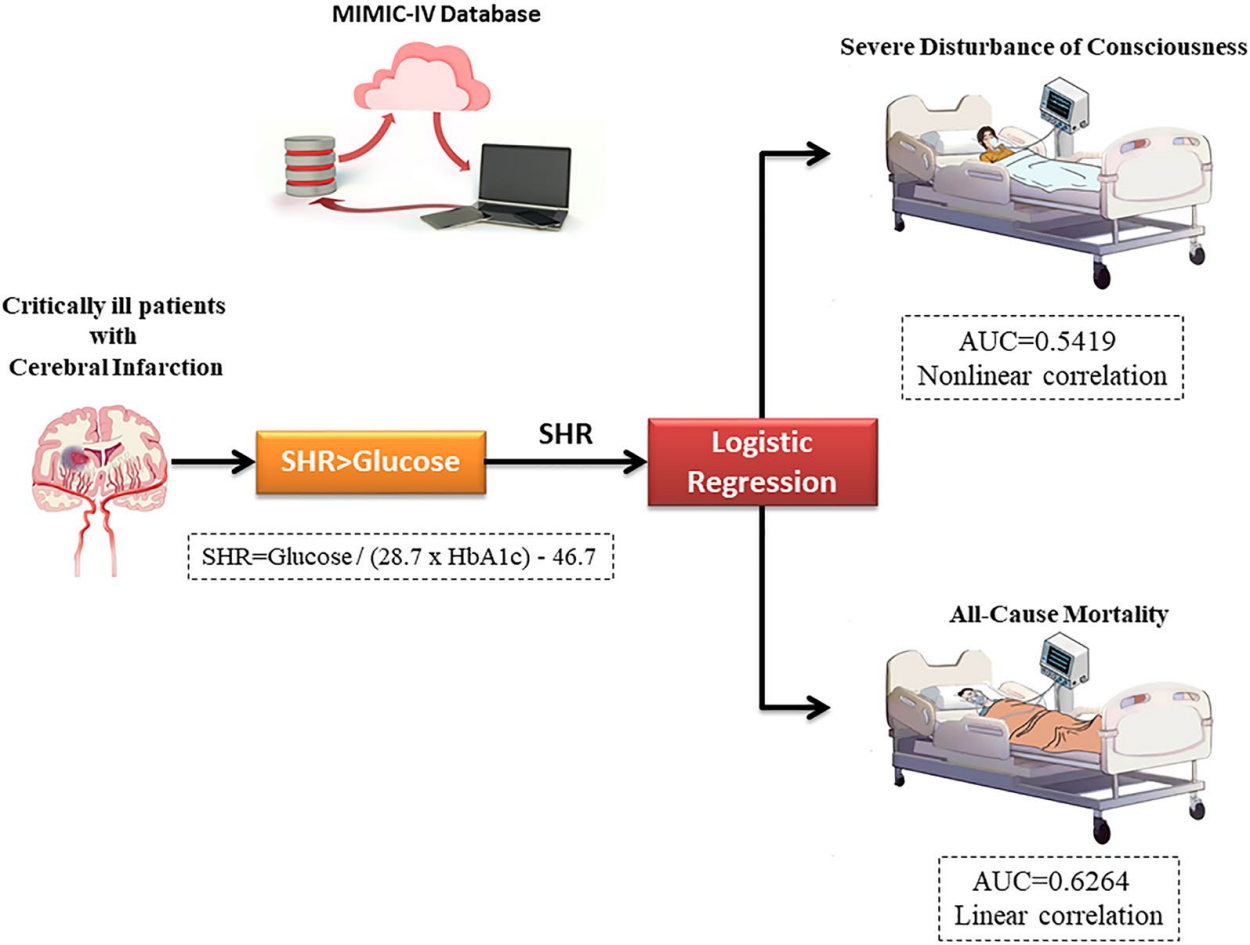

**Supplementary Information:**

The online version contains supplementary material available at 10.1186/s40001-025-02309-9.

## Introduction

Stroke, as a severe ailment, exerts a profound impact on global public health. It not only stands as one of the primary causes of mortality and enduring disabilities but also imposes a substantial medical and socio-economic burden worldwide [1]. Furthermore, ischemic stroke is the most common subtype, accounting for approximately 87% of all stroke cases [2]. Within ischemic stroke, brain cells in the affected area begin to deteriorate due to oxygen and glucose deprivation. Consequently, patients may exhibit various neurological deficits, including disturbances in consciousness, a critical prognostic factor [3]. This condition significantly influences respiratory function, resulting in diverse respiratory impairments and an unfavorable prognosis, marked by elevated incidence and mortality rates [4, 5]. This underscores the pivotal importance of early detection and appropriate management of consciousness disturbances in ischemic stroke patients. Moreover, stroke patients admitted to the Intensive Care Unit (ICU) frequently exhibit a higher prevalence of consciousness disturbances, a more severe clinical condition, and a less favorable prognosis [6].

Stress-induced hyperglycemia (SIH) is characterized by a substantial increase in blood glucose levels, often surpassing 140 mg/dl, which occurs during acute stress situations such as severe infections, surgical procedures, trauma, or the onset of an acute illness, even in individuals with no previous history of diabetes [7]. SIH is a frequently observed clinical phenomenon in ischemic stroke, and its incidence remains notably high [8]. Differentiating between SIH and chronic hyperglycemia in diabetic patients is challenging due to the overlapping increases in blood glucose levels. To address this difficulty, researchers have developed the stress hyperglycemia ratio (SHR) as a specific metric for evaluating and quantifying SIH. It estimates the average blood sugar concentration by comparing the glucose concentration upon patient admission to the level of glycated hemoglobin (HbA1c) [9]. SHR is defined as follows: (admission glucose (mg/ d1)) / (28.7 × HbA1c (%))—46.7. Furthermore, previous studies have established a connection between SHR and short-term as well as long-term mortality rates in critically ill patients [10]. Both single-center and multicenter studies conducted in China have demonstrated that utilizing SHR as an assessment tool allows for a more accurate prediction of in-hospital mortality rates among stroke patients [11–13], as detailed in Supplementary Material. Consequently, we hypothesize that SHR may increase the in-hospital mortality risk for patients with cerebral infarction (CI). However, the association between SHR and severe disturbance of consciousness (DC) in critically ill patients with CI remains ambiguous.

In summary, in order to provide more targeted monitoring and intervention in the early stage for patients with high levels of DC and mortality risk, and to reduce the in-hospital mortality rate and long-term disability risk for patients with CI, the aim of this study is to scrutinize the predictive value of SHR for severe DC and all-cause mortality in critically ill patients diagnosed with CI based on data from the American population study.

## Patients and methods

### Study subject

**Fig. 1:**
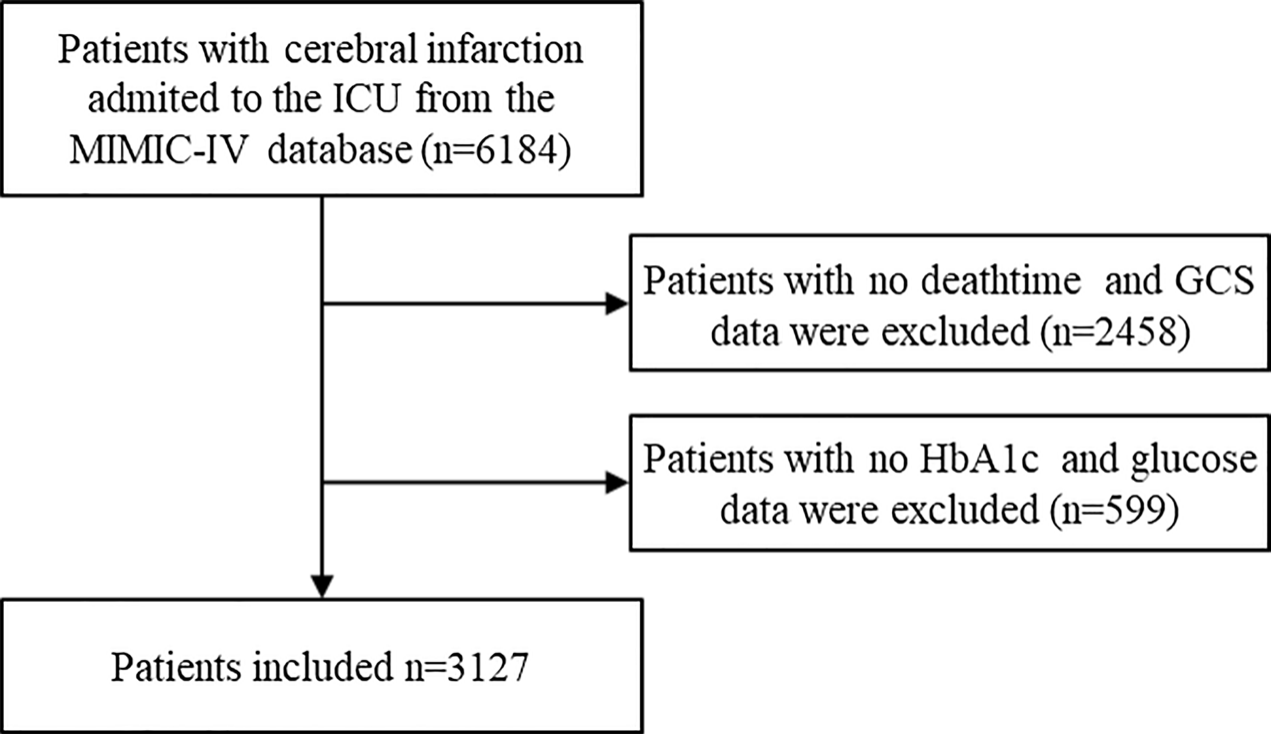
Flowchart for screening

The original data were obtained from the MIMIC-IV database. The MIMIC-IV database is a comprehensive single-center dataset maintained by the MIT Laboratory for Computational Physiology, which primarily includes information from patients admitted to the Emergency Department or Intensive Care Unit at Beth Israel Deaconess Medical Center in Boston, Massachusetts, USA [14]. The author, XiaoSheng Li, has obtained permission to access the dataset (ID: 10,946,391) and is accountable for data extraction. Inclusion criteria were as follows: patients diagnosed with CI based on the International Classification of Diseases, 9th Revision (ICD-9) or International Classification of Diseases, 10th Revision (ICD-10). Exclusion criteria included: (1) missing information on mortality; (2) lack of data on blood sugar levels or hemoglobin [HbA1c] at admission; (3) absence of Glasgow Coma Scale (GCS) scores. In the end, a total of 3,127 critically ill patients with CI were incorporated into this study (Fig. 1).

### Patient characteristics

We extracted various variables from the MIMIC-IV database, encompassing demographics, complications, laboratory results, laboratory indices, reperfusion therapy, medications, and mortality data. To mitigate potential bias stemming from sample exclusion, we calculated the percentage of missing values for each variable. For variables with a missing value proportion of less than 10%, we applied multiple interpolation methods to estimate the missing values. For continuous variables with a missing value proportion exceeding 10%, we opted to exclude the variable from our analysis. In cases where laboratory indicators were assessed multiple times during hospitalization, this study utilized the initial measurement. The variables we included are as follows: demographic information, comprising gender, age, and race; laboratory indicators, encompassing triglycerides, glucose, high-density lipoprotein (HDL), and glycated hemoglobin A1c (HbA1c); complications and personal medical history, including transient ischemic attack (TIA), atrial fibrillation (AF), hypertension, smoking, and alcohol abuse; medications, comprising anticoagulants, antiplatelet drugs, and lipid-lowering drugs; and reperfusion therapy, which includes thrombolysis, angioplasty, and endovascular therapy. Additionally, our analysis incorporated various scoring systems, such as the Glasgow Coma Scale (GCS), Acute Physiology Score III (APSIII), Oxford Acute Severity of Illness Score (OASIS), and Simplified Acute Physiology Score (SAPSII). The calculation of the stress hyperglycemia ratio (SHR) is defined as follows: (admission glucose (mg/ d1)) / (28.7 × HbA1c (%)) – 46.7.

### Outcome measurement

The main outcome measure is the manifestation of severe DC in patients after admission, defined as a GCS score of <  = 8. The secondary outcome indicator is patient mortality during hospitalization.

### Statistical analysis methods

Continuous variables were presented in the form of mean (standard deviation [SD]) or median (quartiles) and were compared using either the Student’s t-test or nonparametric test depending on the specific situation. Categorical variables were displayed as frequency and percentage (%), and inter-group comparisons were conducted utilizing the Pearson Chi-square test or Fisher's exact test. The logistic regression model was utilized to derive odds ratios (ORs) and their 95% confidence intervals (95% CIs). It was also used to adjust for several confounding variables as follows:—Model 1: unadjusted—Model 2: age, gender, and race were adjusted—Model 3: age, gender, race, smoking, alcohol abuse, HDL, and triglycerides were adjusted—Model 4: age, gender, race, smoking, alcohol abuse, HDL, triglycerides, TIA, AF, hypertension, anticoagulants, antiplatelet drugs, lipid-lowering drugs, thrombolysis, angioplasty, and mechanical embolectomy were adjusted. In this study, the restricted cubic spline (RCS) method was employed to explore the potential non-linear association between levels of the SHR index and outcomes. To compare the predictive ability of the SHR index and admission blood sugar level, we computed the area under the ROC curve (AUC) separately. Additionally, we plotted clinical decision curves and calculated the integrated discrimination improvement (IDI) to scrutinize the improvement in predictive ability and clinical value after incorporating the SHR index into the scoring tool. A positive value suggests positive improvement, while a negative value suggests negative improvement. The statistical significance criterion was a *P*-value < 0.05. The statistical analysis for this study was carried out utilizing R software version 4.3.2 (R Core Team. (2023). R: A Language and Environment for Statistical Computing. R Foundation for Statistical Computing, Vienna, Austria. URL https://www.R-project.org/).

## Results

### Baseline characteristics

This study involved 3,127 critically ill patients with CI. Among them, 850 (27.1%) patients experienced severe DC, and 202 (6.46%) patients passed away in the hospital. The median age of the patients was 70 years old, with a range of [59.00, 80.00]. There were 1569 (50.2%) males and 1558 (49.8%) females. The median standardized hospital readmission (SHR) of all patients with ischemic stroke was 0.97, with a range of [0.82, 1.18]. The median SHR of patients who died was 1.10, with a range of [0.93, 1.38]. The median SHR of patients with severe DC was 1.00, with a range of [0.82, 1.26]. Disparities were noted between the two groups with and without in-hospital mortality in terms of SHR, age, HDL, race, TIA, anticoagulants, lipid-lowering drugs, antiplatelet drugs, and smoking (*p* < 0.05). Additionally, differences were noted between the two groups with and without severe DC in terms of SHR, HDL, race, TIA, AF, antiplatelet drugs, lipid-lowering drugs, and alcohol abuse (*p* < 0.05). (Specific information is presented in Tables 1, 2.)

**Table 1:** Baseline information on the severity of disturbance of consciousness of patients with cerebral infarction (GCS <  = 8 indicates the occurrence of severe disturbance of consciousness during hospitalization)

	Level	Overall	GCS > 8	GCS < = 8	*P*
*N*		3127	2277	850	
Anchor_age (median [IQR])		70.00 [59.00, 80.00]	70.00 [59.00, 80.00]	71.00 [59.00, 81.00]	0.499
HDL (median [IQR])		46.00 [36.00, 58.00]	46.00 [37.00, 59.00]	44.00 [34.00, 57.00]	< 0.001
Triglycerides (median [IQR])		115.00 [83.00, 163.00]	116.00 [83.00, 162.00]	113.00 [82.00, 165.00]	0.782
SHR (median [IQR])		0.97 [0.82, 1.18]	0.96 [0.82, 1.15]	1.00 [0.82, 1.26]	< 0.001
Gender (%)	"MALE"	1569 (50.2)	1135 (49.8)	434 (51.1)	0.573
	"FEMALE"	1558 (49.8)	1142 (50.2)	416 (48.9)	
Race (%)	"UNKNOWN"	433 (13.8)	293 (12.9)	140 (16.5)	0.001
	"WHITE"	2077 (66.4)	1564 (68.7)	513 (60.4)	
	"BLACK"	419 (13.4)	288 (12.6)	131 (15.4)	
	"ASIAN"	90 (2.9)	60 (2.6)	30 (3.5)	
	"OTHER"	108 (3.5)	72 (3.2)	36 (4.2)	
TIA (%)	"NO"	2002 (64.0)	1423 (62.5)	579 (68.1)	0.004
	"YES"	1125 (36.0)	854 (37.5)	271 (31.9)	
Atrial fibrillation (%)	"NO"	1725 (55.2)	1282 (56.3)	443 (52.1)	0.04
	"YES"	1402 (44.8)	995 (43.7)	407 (47.9)	
Hypertension (%)	"NO"	974 (31.1)	712 (31.3)	262 (30.8)	0.845
	"YES"	2153 (68.9)	1565 (68.7)	588 (69.2)	
Anticoagulation (%)	"NO"	145 (4.6)	102 (4.5)	43 (5.1)	0.555
	"YES"	2982 (95.4)	2175 (95.5)	807 (94.9)	
Antiplatelet (%)	"NO"	378 (12.1)	255 (11.2)	123 (14.5)	0.015
	"YES"	2749 (87.9)	2022 (88.8)	727 (85.5)	
Lipid-lowering (%)	"NO"	607 (19.4)	379 (16.6)	228 (26.8)	< 0.001
	"YES"	2520 (80.6)	1898 (83.4)	622 (73.2)	
Alcohol (%)	"NO"	3004 (96.1)	2180 (95.7)	824 (96.9)	0.152
	"YES"	123 (3.9)	97 (4.3)	26 (3.1)	
Smoking (%)	"NO"	2518 (80.5)	1840 (80.8)	678 (79.8)	0.545
	"YES"	609 (19.5)	437 (19.2)	172 (20.2)	
Angioplasty (%)	"NO"	3078 (98.4)	2242 (98.5)	836 (98.4)	0.953
	"YES"	49 (1.6)	35 (1.5)	14 (1.6)	
Endovascular_treatment (%)	"NO"	2960 (94.7)	2153 (94.6)	807 (94.9)	0.735
	"YES"	167 (5.3)	124 (5.4)	43 (5.1)	
Thrombolytic (%)	"NO"	2791 (89.3)	2026 (89.0)	765 (90.0)	0.449
	"YES"	336 (10.7)	251 (11.0)	85 (10.0)

**Table 2:** Baseline information on in-hospital mortality of patients with cerebral infarction

	Level	Overall	Survival	Death	*P*
*N*		3127	2925	202	
Anchor_age (median [IQR])		70.00 [59.00, 80.00]	70.00 [59.00, 80.00]	76.00 [65.00, 85.00]	< 0.001
HDL (median [IQR])		46.00 [36.00, 58.00]	46.00 [36.00, 58.00]	41.50 [33.00, 55.00]	0.001
Triglycerides (median [IQR])		115.00 [83.00, 163.00]	115.00 [83.00, 162.00]	115.00 [81.25, 170.75]	0.829
SHR (median [IQR])		0.97 [0.82, 1.18]	0.96 [0.82, 1.16]	1.10 [0.93, 1.38]	< 0.001
Gender (%)	"MALE"	1569 (50.2)	1466 (50.1)	103 (51.0)	0.868
	"FEMALE"	1558 (49.8)	1459 (49.9)	99 (49.0)	
Race (%)	"UNKNOWN"	433 (13.8)	354 (12.1)	79 (39.1)	< 0.001
	"WHITE"	2077 (66.4)	1982 (67.8)	95 (47.0)	
	"BLACK"	419 (13.4)	403 (13.8)	16 (7.9)	
	"ASIAN"	90 (2.9)	84 (2.9)	6 (3.0)	
	"OTHER"	108 (3.5)	102 (3.5)	6 (3.0)	
TIA (%)	"NO"	2002 (64.0)	1824 (62.4)	178 (88.1)	< 0.001
	"YES"	1125 (36.0)	1101 (37.6)	24 (11.9)	
Atrial fibrillation (%)	"NO"	1725 (55.2)	1614 (55.2)	111 (55.0)	1
	"YES"	1402 (44.8)	1311 (44.8)	91 (45.0)	
Hypertension (%)	"NO"	974 (31.1)	900 (30.8)	74 (36.6)	0.096
	"YES"	2153 (68.9)	2025 (69.2)	128 (63.4)	
Anticoagulation (%)	"NO"	145 (4.6)	100 (3.4)	45 (22.3)	< 0.001
	"YES"	2982 (95.4)	2825 (96.6)	157 (77.7)	
Antiplatelet (%)	"NO"	378 (12.1)	309 (10.6)	69 (34.2)	< 0.001
	"YES"	2749 (87.9)	2616 (89.4)	133 (65.8)	
Lipid-lowering (%)	"NO"	607 (19.4)	509 (17.4)	98 (48.5)	< 0.001
	"YES"	2520 (80.6)	2416 (82.6)	104 (51.5)	
Alcohol (%)	"NO"	3004 (96.1)	2805 (95.9)	199 (98.5)	0.096
	"YES"	123 (3.9)	120 (4.1)	3 (1.5)	
Smoking (%)	"NO"	2518 (80.5)	2336 (79.9)	182 (90.1)	0.001
	"YES"	609 (19.5)	589 (20.1)	20 (9.9)	
Angioplasty (%)	"NO"	3078 (98.4)	2882 (98.5)	196 (97.0)	0.171
	"YES"	49 (1.6)	43 (1.5)	6 (3.0)	
Endovascular_treatment (%)	"NO"	2960 (94.7)	2771 (94.7)	189 (93.6)	0.58
	"YES"	167 (5.3)	154 (5.3)	13 (6.4)	
Thrombolytic (%)	"NO"	2791 (89.3)	2611 (89.3)	180 (89.1)	1
	"YES"	336 (10.7)	314 (10.7)	22 (10.9)	

### The influence of the SHR index on the severity of DC and in-hospital mortality

SHR index on the severity of DC indicated that model 1 (unadjusted), model 2 (baseline demographic statistics were adjusted), and model 3 (baseline demographic statistics were adjusted, and laboratory indicators were added) all showed that the SHR index was a notable risk factor for the severity of DC in patients with CI (model 1: OR = 1.639, 95% CI = 1.351–1.994, *p* < 0.001; model 2: OR = 1.635, 95% CI = 1.348–1.991, *p* < 0.001; model 3: OR = 1.621, 95% CI = 1.334–1.997, *p* < 0.001). Even after full adjustment (model 4), the SHR index remained an independent predictive factor for the risk of DC in patients with CI, with an increasing risk as the SHR value increased (OR = 1.558, 95% CI = 1.279–1.905, *p* < 0.001). (Details are presented in Table 3.)

**Table 3:** Logistic regression analysis of disturbance of consciousness and in-hospital mortality of patients with cerebral infarction (model 1: unadjusted; model 2: age, gender, and race were adjusted; model 3: age, gender, race, smoking, alcohol abuse, HDL, and triglycerides were adjusted; model 4: age, gender, race, smoking, alcohol abuse, HDL, triglycerides, TIA, AF, hypertension, anticoagulants, antiplatelet drugs, lipid-lowering drugs, thrombolysis, angioplasty, and mechanical embolectomy were adjusted; GCS <  = 8 indicates the occurrence of severe disturbance of consciousness during hospitalization.)

	Model1				Model2				Model3				Model4			
	OR	OR(95%CI)		*P*	OR	OR(95%CI)		*P*	OR	OR(95%CI)		*P*	OR	OR(95%CI)		*P*
GCS < = 8	1.639	1.351	1.994	< 0.001	1.635	1.348	1.991	< 0.001	1.621	1.334	1.997	< 0.001	1.558	1.279	1.905	< 0.001
Death	2.042	1.563	2.666	< 0.001	2.197	1.649	2.906	< 0.001	2.106	1.574	2.806	< 0.001	1.905	1.363	2.620	< 0.001

The analysis of the SHR index on in-hospital mortality demonstrated that the SHR index served as an independent predictor of the risk of DC of patients with CI whether in the unadjusted model (model 1) or in the model with gradually increasing adjusted variables (models 2 to 4) (model 1: 2.042 (95% CI = 1.563–2.666, *p* < 0.001; model 2: OR = 2.197 (95% CI = 1.649–2.906, *p* < 0.001); model 3: OR = 2.106 (95% CI = 1.574–2.806, *p* < 0.001); model 4: OR = 1.905 (95% CI = 1.363–2.620, *p* < 0.001)). As the SHR index increased, the risk of in-hospital mortality continued to rise. (It is illustrated in Table 3.)

### Nonlinear association between SHR and severe DC and in-hospital mortality

**Fig. 2:**
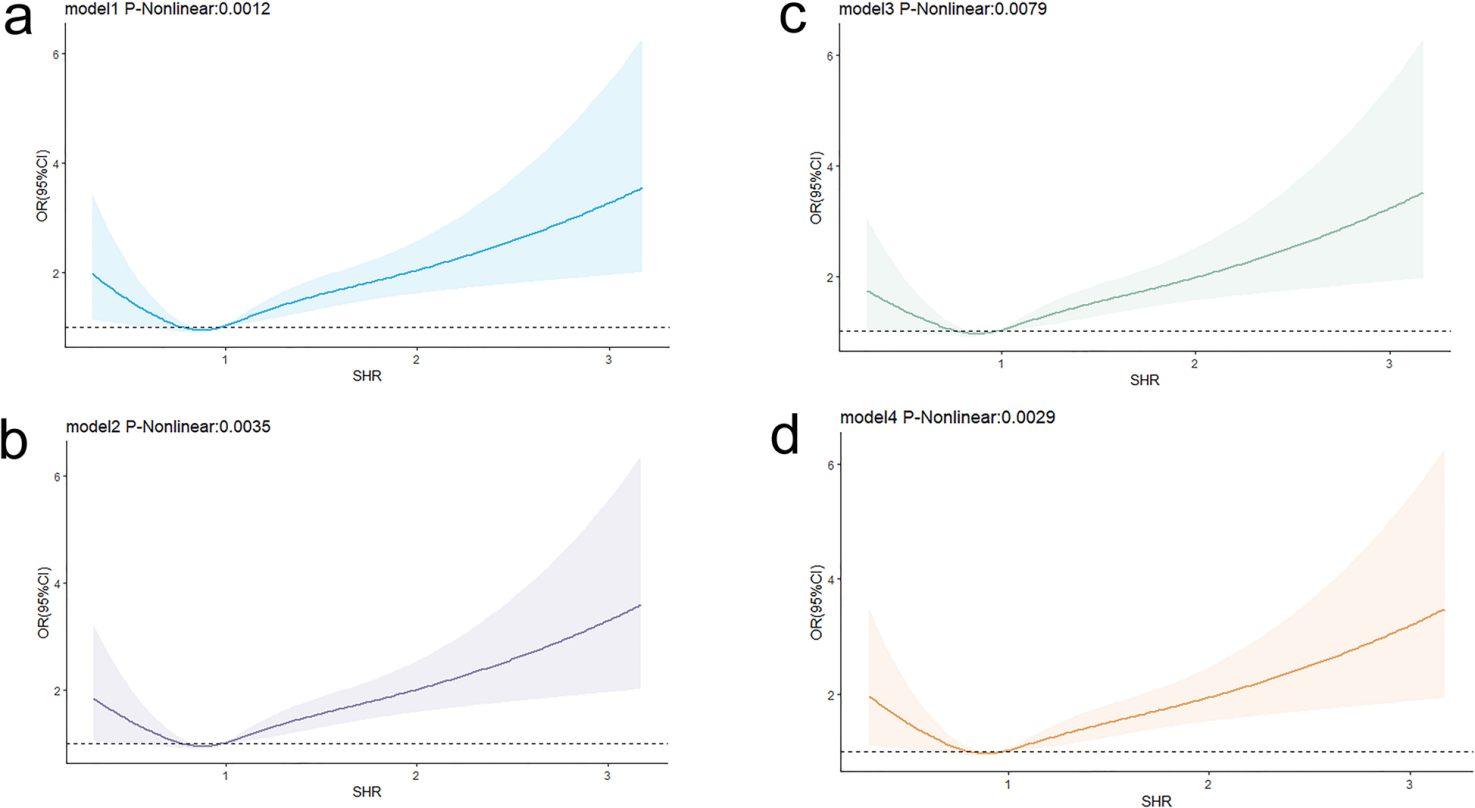
RCS curve of SHR index and OR of patients with cerebral infarction:** a**,** b**,** c**,** d** RCS curve of severe disturbance of consciousness

**Fig. 3:**
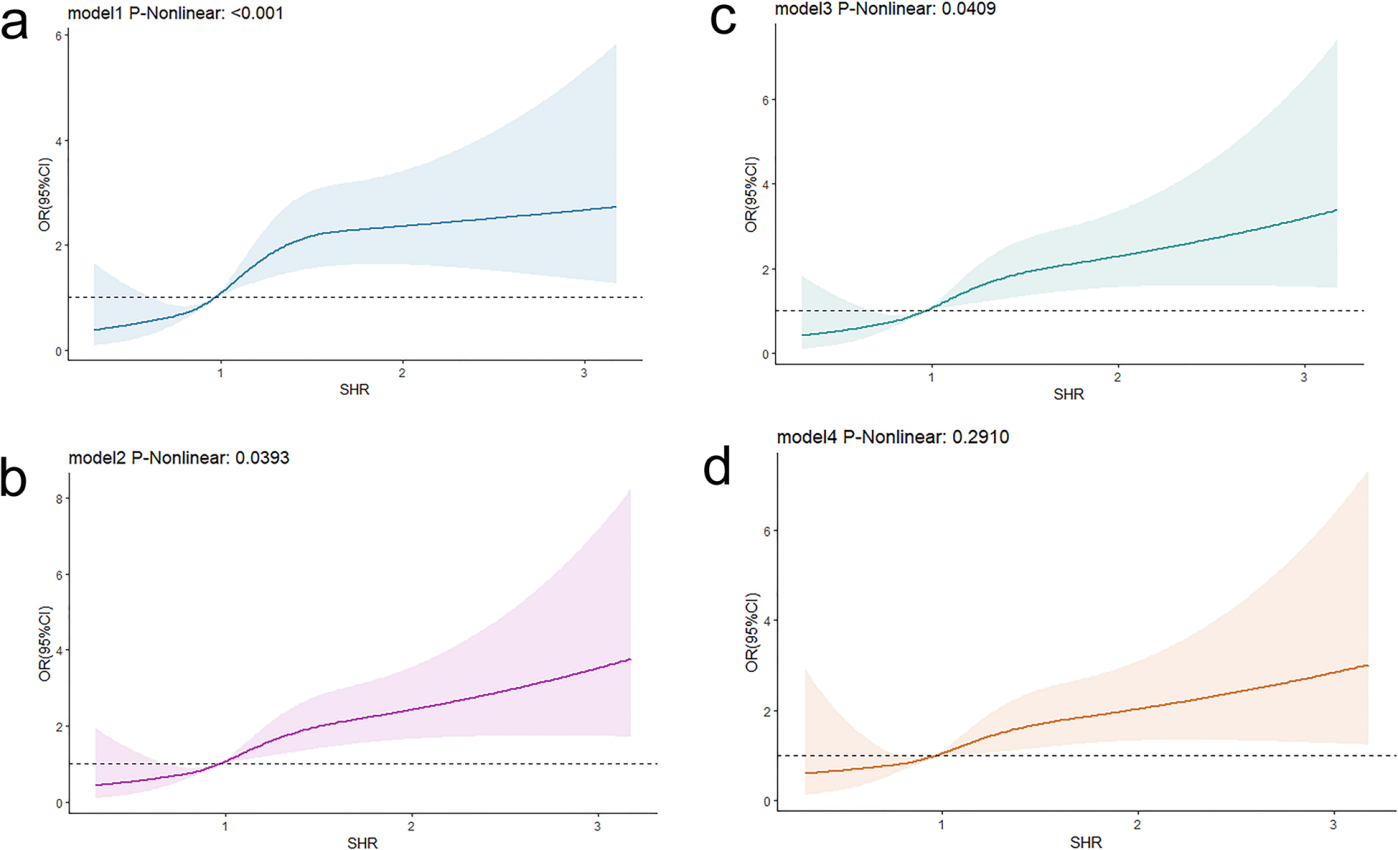
RCS curve of SHR index and OR of patients with cerebral infarction:** a**,** b**,** c**,** d** RCS curve of in-hospital mortality

We utilized restricted cubic spline (RCS) curves to assess the non-linear association between the SHR index and its association with outcomes. The findings indicate a non-linear association between SHR and severe DC, whereas a linear correlation was observed with in-hospital mortality. When evaluating the link between SHR and severe DC in patients with CI, all models demonstrated a significant non-linear relationship (model 1: P-nonlinear = 0.0012; model 2: P-nonlinear = 0.0035; model 3: P-nonlinear = 0.0079; model 4: P-nonlinear = 0.0029). After comprehensive adjustment (model 4), it became apparent that as the SHR value decreased below 0.796, the risk of patients experiencing severe DC gradually decreased. Conversely, when the SHR value exceeded 0.796, the risk was gradually increasing (as depicted in Fig. 2). Furthermore, when exploring the association between SHR and in-hospital mortality, the unadjusted model (model 1) revealed a significant non-linear relationship (model 1: P-nonlinear < 0.001). However, with the gradual adjustment of confounding factors, SHR exhibited a linear relationship with the risk of in-hospital mortality (model 4: P-nonlinear = 0.2910) (as illustrated in Fig. 3).

### Prediction ability of the SHR index and admission blood sugar level

**Fig. 4:**
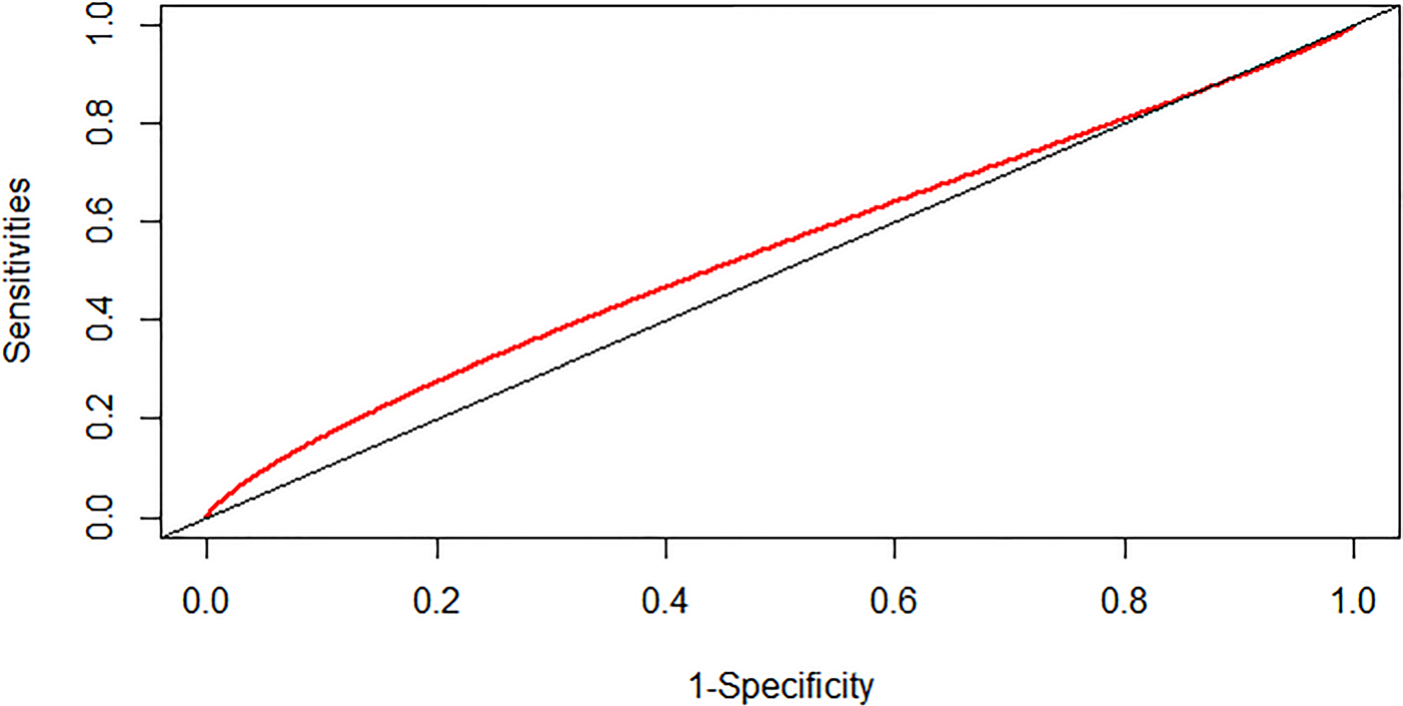
ROC curve analysis of the SHR index for predicting severe disturbance of consciousness of patients with cerebral infarction

**Fig. 5:**
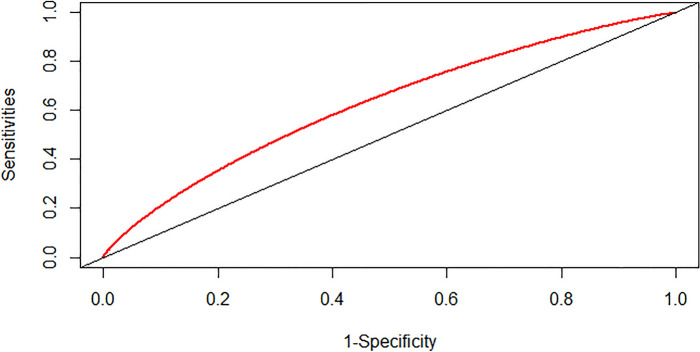
ROC curve analysis of the SHR index for predicting in-hospital mortality of patients with cerebral infarction

**Fig. 6:**
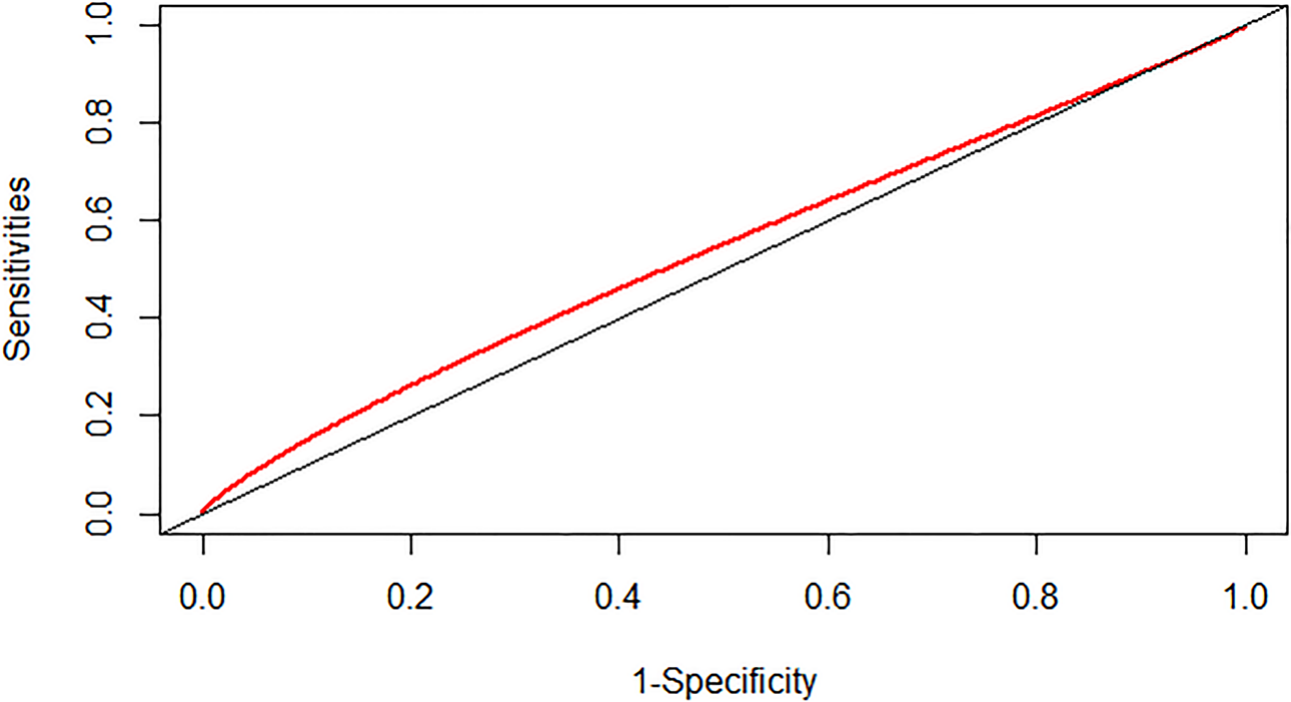
ROC Curve analysis of the SHR index for predicting severe disturbance of consciousness of patients with cerebral infarction

**Fig. 7:**
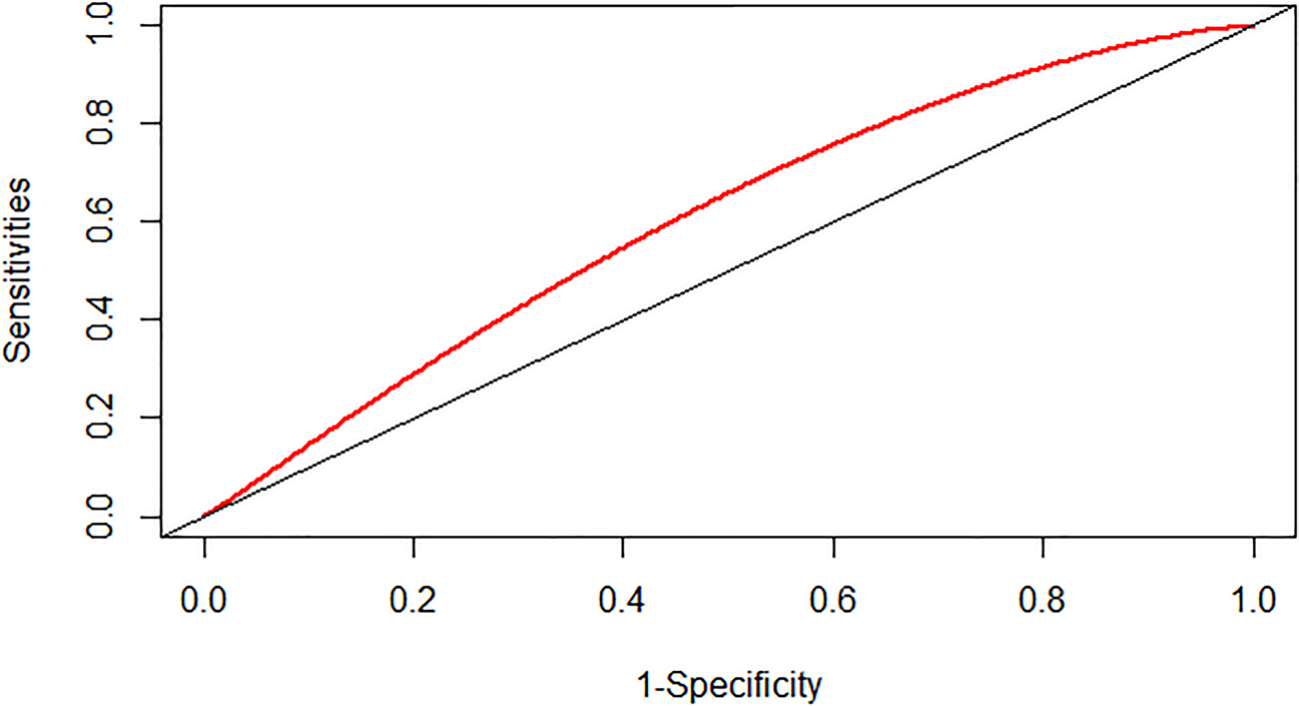
ROC curve analysis of the SHR index for predicting in-hospital mortality of patients with cerebral infarction

We assessed the predictive capacity of the SHR index and admission blood sugar level for the risk of severe DC and in-hospital mortality in patients with CI by calculating the area under the ROC curve (AUC). The findings indicated that the SHR index displayed a moderate predictive ability for severe DC in patients with CI, with an AUC value of 0.5419 (95% CI: 0.5188–0.5661) (as depicted in Fig. 4). The SHR index exhibited superior performance in predicting the risk of in-hospital mortality in patients with CI, with an AUC value of 0.6264 (95% CI: 0.5881–0.6662) (as illustrated in Fig. 5). However, the predictive ability of admission blood sugar level for the risk of severe DC and in-hospital mortality in patients with CI was slightly lower compared to the SHR index. The AUC value for predicting severe DC was 0.5387 (95% CI: 0.5147–0.5669) (as shown in Fig. 6), and the AUC value for forecasting in-hospital mortality was 0.6058 (95% CI: 0.5649–0.6457) (as illustrated in Fig. 7).

### The incremental effect of the SHR index

**Fig. 8:**
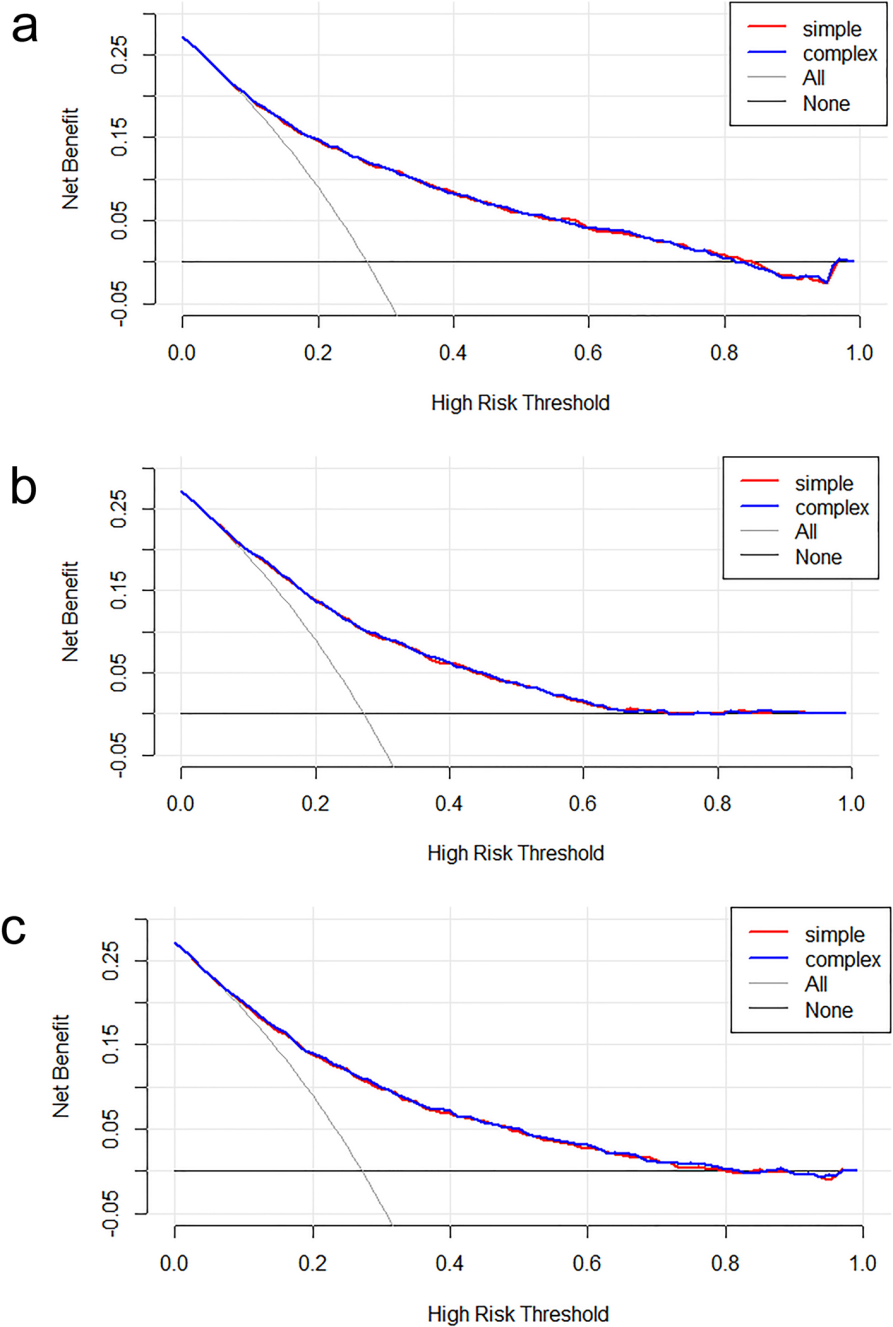
Decision curve analysis of the SHR index for predicting the severe disturbance of consciousness of patients with cerebral infarction: **a** GCS ~ apsiii; **b** GCS ~ oasis; **c** GCS ~ sapsii

**Fig. 9:**
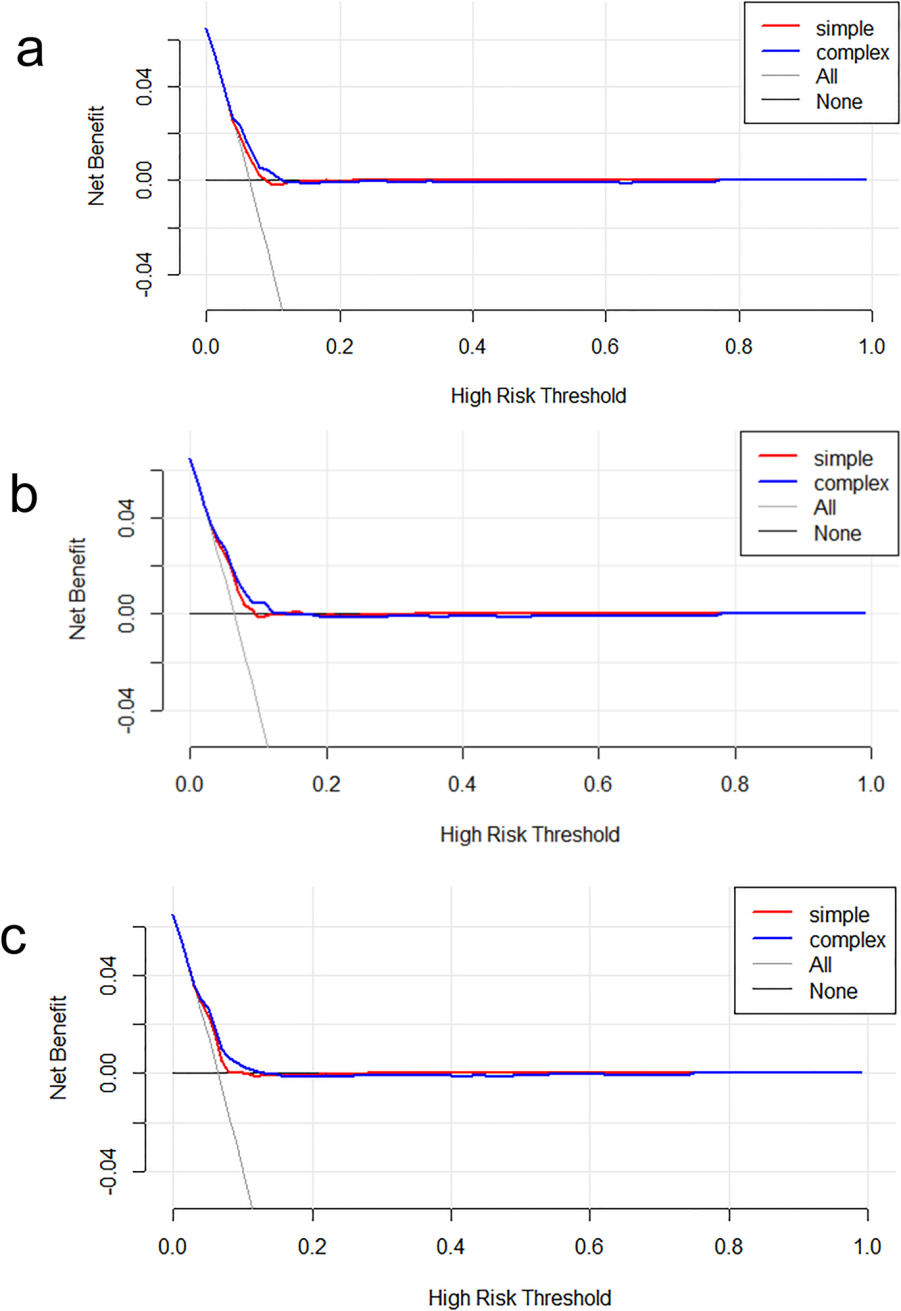
Decision curve analysis of the SHR index for predicting the in-hospital mortality of patients with cerebral infarction: **a** status ~ apsiii; **b** status ~ oasis; **c** status ~ sapsii

When evaluating the SHR index, we computed the IDI for the scoring tools (APSIII, OASIS, SAPSII) and analyzed the impact of the SHR index on their predictive capabilities. This study revealed that when forecasting severe DC in patients with CI, the SHR index significantly enhanced the incremental improvement of SAPSII score (*P* < 0.01). However, no statistically notable incremental improvement was noted for APSIII and OASIS scores. (*P* > 0.05) (Table 4). Conversely, when considering the SHR index, all scoring tools significantly improved their predictive accuracy for in-hospital mortality (*P* < 0.05) (Table 4). Furthermore, the findings suggested that the net clinical benefits of each scoring tool also increased when taking the SHR index into account, as shown in the clinical decision curve (Figs. 8, 9).

**Table 4:** Incremental effects of the SHR index on severe disturbance of consciousness and in-hospital mortality of patients with cerebral infarction (GCS <  = 8 indicates the occurrence of severe disturbance of consciousness during hospitalization)

Score	GCS < = 8		Death	
	IDI [95% CI]	*p*	IDI [95% CI]	*P*
Apsiii	[95% CI]: 0.0012 [− 0.0004–0.0027]	0.14183	[95% CI]: 0.0063 [0.0027–0.0099]	0.00056
Oasis	[95% CI]: 0.0016 [− 0.0002–0.0035]	0.07629	[95% CI]: 0.0051 [0.0017–0.0085]	0.00351
Sapsii	[95% CI]: 0.0043 [0.0014–0.0073]	0.00421	[95% CI]: 0.0068 [0.0031–0.0105]	0.00032

## Discussion

This study marks the inaugural exploration of the effectiveness of the SHR index in foreseeing severe disturbances of consciousness in critically ill patients afflicted with CI. Furthermore, it underscores the significance of utilizing the SHR based on the American population to predict all-cause mortality in patients with CI. The findings have revealed that an elevated SHR index level serves as an independent risk factor significantly linked to severe DC and in-hospital mortality in the critically ill population with cerebrovascular disease. Additionally, it has been noted that a non-linear relationship exists between SHR and the risk of severe DC, whereas a linear relationship exists concerning all-cause mortality.

When compared to admission blood sugar levels, the SHR stands out as a more effective indicator in reflecting the association between SIH and adverse event outcomes in patients with CI. This study has demonstrated that SIH is closely linked not only to the severity of ischemic stroke [15], but also to poor prognosis within 90 days after the stroke [16]. Furthermore, an increased SIH index in ischemic stroke patients is correlated with a heightened risk of adverse events during reperfusion therapy, particularly notable in non-diabetic patient populations [17–20]. Additionally, this index significantly increases the risk of unfavorable prognosis in patients with acute myocardial infarction [21, 22]. Additionally, meta-analyses have demonstrated that SHR is linked to a higher risk of all-cause mortality after both myocardial infarction and ischemic stroke [23]. In the assessment of a patient's blood sugar level control status, the stress hyperglycemia ratio (SHR) plays a pivotal role. It is important to note that there are physiological distinctions between chronic hyperglycemia and SIH [24, 25]. Therefore, SHR provides a more comprehensive perspective, effectively reflecting the association between SIH and adverse events in patients. Tziomalos K et al. utilized admission glucose levels as an indicator to explore the potential association between SIH and adverse events in patients with ischemic stroke [26]. Their findings suggest no significant association between SIH and poor prognosis in ischemic stroke patients. In contrast, Zhu B et al. used SHR as the evaluation standard [17], and their study results demonstrated an association between SIH and adverse event outcomes in ischemic stroke. Furthermore, our study revealed that admission blood sugar levels had a weaker predictive ability than the SHR index in forecasting the risk of severe DC and in-hospital mortality in patients with CI. Therefore, there may be limitations in using a single blood glucose level as an SIH evaluation indicator for predicting poor prognosis in patients with ischemic stroke.

While this study has demonstrated the effectiveness of the SHR index in predicting the occurrence of severe DC and all-cause mortality in critically ill patients with CI, the link between the prognosis of CI patients and SIH remains not entirely clarified. This may be related to the impact of SIH on the circulatory system, reduced cerebral energy metabolism, induction of cellular toxicity and cell death, and the release of inflammatory cytokines [27]. SIH can lead to transient vasoconstriction of small arteries [28], which can be achieved through the activity of L-type Ca^2+^ channels and the PKA-dependent regulatory effect on vascular reactivity [29]. This vasoconstriction results in a decrease in cerebral blood flow, thereby exacerbating brain tissue hypoxia. Under aerobic conditions, the human body primarily metabolizes glucose and generates reactive oxygen species (ROS) through the action of mitochondrial and microsomal oxidative enzymes, as well as cytoplasmic pro-oxidant enzymes. Accumulation of ROS can trigger oxidative stress, hyperglycemia, and insulin resistance [30]. While the decrease in mitochondrial respiration and the increase in ROS are not always pathological [31], in the case of SIH, the increased ROS accumulation can lead to oxidative stress and progressive insulin resistance. The imbalance between intracellular pro-oxidants and antioxidants results in elevated ROS levels, exacerbating oxidative stress, hyperglycemia, and inflammation. Hyperglycemia exacerbates glucose metabolism through protein kinase C, polyol, and hexosamine pathways, promoting glucose auto-oxidation and the formation of advanced glycation end products (AGEs). These AGEs interact with their receptor (RAGE), disrupting cell structure, leading to increased oxidative stress and further deterioration of hyperglycemia [32]. The blood–brain barrier (BBB) consists of endothelial cells, which typically prevent most substances from freely crossing, maintaining a stable brain environment. Damage to the BBB can allow harmful substances to invade the brain, worsening neuroinflammation and neuronal damage [33]. SIH can increase ROS production and oxidative stress, activating inflammatory pathways, such as the release of pro-inflammatory cytokines, collectively disrupting BBB integrity and function [34, 35]. For instance, the mediating effect of peroxynitrite anions can activate MMPs and NLRP3 inflammasomes, exacerbating BBB damage and hemorrhagic transformation, and leading to a poorer prognosis in hyperglycemic ischemic stroke patients [36]. Additionally, in an animal experiment, it was found that S-nitrosoglutathione (GSNO) seems to be one of the causes of severe DC in critically ill patients with CI under SIH [37]. This discovery provides new insights into the risk of high CI and severe DC caused by SIH, and it is expected to be further verified in future clinical studies.

### Clinical recommendations

Maintaining moderate hyperglycemia seems to be beneficial for the prognosis of critically ill patients with CI. This study has revealed a non-linear relationship between the severity of DC in patients with CI and the SHR index, aligning with earlier observations [38]. Specifically, there exists a "J-shaped" dose–response relationship between SHR and the poor prognosis of patients with CI. Consequently, regulating SHR at a reasonable level may hold the potential to offer protection in the prognosis of critically ill patients with CI. Moreover, in accordance with the Acute Stroke Treatment Guidelines released by the American Heart Association/American Stroke Association (AHA/ASA), it is recommended to maintain glucose levels within the range of 7.8–10.0 mmol/L for ischemic stroke patients experiencing SIH [39]. This recommended blood sugar level is notably higher than the fasting blood glucose control standards recommended by the American Diabetes Association (ADA) for non-diabetic adults (3.9–5.5 mmol/L) [40] as well as the fasting blood glucose control recommendations for diabetic patients (4.4–7.2 mmol/L) [41]. This contrast highlights the potential positive role of moderately elevated glucose levels in reducing cerebral ischemic damage in critically ill patients with CI. Another significant finding of this study is that SHR appears to be more effective in reflecting the association between SIH and adverse event outcomes in patients with CI. Consequently, in future studies, exploring the appropriate range of moderate SHR index as an alternative indicator for blood sugar level control in critically ill patients with CI will hold important value.

### Limitations

This study used the MIMIC database to explore the use of SHR as a predictive indicator for severe DC and all-cause mortality in critically ill patients with CI. However, this study had certain limitations. Dependence on data from a single source may impact the generalizability of the results. Therefore, in future studies, multicenter data are still needed for validation, and the applicability and effectiveness of SHR in different types of vascular diseases should be explored.

## Conclusions

Our study shows that SHR is an important predictive indicator for severe DC and all-cause mortality in critically ill patients with CI. It has a non-linear relationship with the risk of severe DC and a linear relationship with all-cause mortality. Future studies should further explore the application of the SHR index in clinical practice to optimize the management and prognosis of patients with CI.

## Supplementary Information

Supplementary Material 1. Clinical Advances in the Association Between SHR and Stroke: A Five-Year Review

## Data Availability

No datasets were generated or analysed during the current study.

## Abbreviations

SHR: Stress hyperglycemia ratio
DC: Disturbance of consciousness
ROC: Receiver operating characteristic
ICU: Intensive care unit
SIH: Stress-induced hyperglycemia
HbA1c: Glycated hemoglobin A1c
CI: Cerebral infarction
ICD-9: International Classification of Diseases, 9th Revision
ICD-10: International Classification of Diseases, 10th Revision
GCS: Glasgow Coma Scale
HDL: High-density lipoprotein
TIA: Transient ischemic attack
AF: Atrial fibrillation
GCS: Glasgow Coma Scale
APSIII: Acute Physiology Score III
OASIS: Oxford Acute Severity of Illness Score
SAPSII: Simplified Acute Physiology Score
ORs: Odds ratios
RCS: Restricted cubic spline
AUC: Area under the ROC curve
IDI: Integrated discrimination improvement
AHA/ASA: American Heart Association/American Stroke Association
ADA: American Diabetes Association
ROS: Reactive oxygen species
AGEs: Advanced glycation end products
RAGE: Receptor for advanced glycation end products
BBB: Blood–brain barrier
GSNO: S-Nitrosoglutathione

## References

[1] Roth GA, Mensah GA, Johnson CO, Addolorato G, Ammirati E, Baddour LM, et al. Global burden of cardiovascular diseases and risk factors, 1990–2019: update from the GBD 2019 Study. J Am Coll Cardiol. 2020;76(25):2982–3021. 33309175 10.1016/j.jacc.2020.11.010 PMC7755038

[2] Benjamin EJ, Blaha MJ, Chiuve SE, Cushman M, Das SR, Deo R, et al. Heart Disease and Stroke Statistics-2017 update: a report from the American Heart Association. Circulation. 2017;135(10):e146–603. 28122885 10.1161/CIR.0000000000000485 PMC5408160

[3] Stinear CM. Prediction of motor recovery after stroke: advances in biomarkers. Lancet Neurol. 2017;16(10):826–36. 28920888 10.1016/S1474-4422(17)30283-1

[4] Emsley HC, Hopkins SJ. Acute ischaemic stroke and infection: recent and emerging concepts. Lancet Neurol. 2008;7(4):341–53. 18339349 10.1016/S1474-4422(08)70061-9

[5] McCrea MA, Giacino JT, Barber J, Temkin NR, Nelson LD, Levin HS, et al. Functional outcomes over the first year after moderate to severe traumatic brain injury in the prospective, longitudinal TRACK-TBI study. JAMA Neurol. 2021;78(8):982–92. 34228047 10.1001/jamaneurol.2021.2043 PMC8261688

[6] Chang CWJ, Provencio JJ, Shah S. Neurological critical care: the evolution of cerebrovascular critical care. Crit Care Med. 2021;49(6):881–900. 33653976 10.1097/CCM.0000000000004933

[7] Harp JB, Yancopoulos GD, Gromada J. Glucagon orchestrates stress-induced hyperglycaemia. Diabetes Obes Metab. 2016;18(7):648–53. 27027662 10.1111/dom.12668 PMC5084782

[8] Zhang H, Yue K, Jiang Z, Wu X, Li X, Luo P, et al. Incidence of stress-induced hyperglycemia in acute ischemic stroke: a systematic review and meta-analysis. Brain Sci. 2023;13(4). 10.3390/brainsci13040556 PMC10136900 37190521

[9] Roberts GW, Quinn SJ, Valentine N, Alhawassi T, O’Dea H, Stranks SN, et al. Relative hyperglycemia, a marker of critical illness: introducing the stress hyperglycemia ratio. J Clin Endocrinol Metab. 2015;100(12):4490–7. 26485219 10.1210/jc.2015-2660

[10] Li L, Zhao M, Zhang Z, Zhou L, Zhang Z, Xiong Y, et al. Prognostic significance of the stress hyperglycemia ratio in critically ill patients. Cardiovasc Diabetol. 2023;22(1):275. 37833697 10.1186/s12933-023-02005-0 PMC10576399

[11] Zhu B, Pan Y, Jing J, Meng X, Zhao X, Liu L, et al. Stress hyperglycemia and outcome of non-diabetic patients after acute ischemic stroke. Front Neurol. 2019;10:1003. 31620074 10.3389/fneur.2019.01003 PMC6759951

[12] Mi D, Li Z, Gu H, Jiang Y, Zhao X, Wang Y, et al. Stress hyperglycemia is associated with in-hospital mortality in patients with diabetes and acute ischemic stroke. CNS Neurosci Ther. 2022;28(3):372–81. 35084107 10.1111/cns.13764 PMC8841306

[13] Yang CJ, Liao WI, Wang JC, Tsai CL, Lee JT, Peng GS, et al. Usefulness of glycated hemoglobin A1c-based adjusted glycemic variables in diabetic patients presenting with acute ischemic stroke. Am J Emerg Med. 2017;35(9):1240–6. 28363620 10.1016/j.ajem.2017.03.049

[14] Johnson AEW, Bulgarelli L, Shen L, Gayles A, Shammout A, Horng S, et al. MIMIC-IV, a freely accessible electronic health record dataset. Sci Data. 2023;10(1):1. 36596836 10.1038/s41597-022-01899-x PMC9810617

[15] Liu C, Zhu XP, Zhu XW, Jiang YM, Xi GJ, Xu L. The acute-to-chronic glycemic ratio correlates with the severity of illness at admission in patients with diabetes experiencing acute ischemic stroke. Front Neurol. 2022;13: 938612. 36419531 10.3389/fneur.2022.938612 PMC9676263

[16] Peng Z, Song J, Li L, Guo C, Yang J, Kong W, et al. Association between stress hyperglycemia and outcomes in patients with acute ischemic stroke due to large vessel occlusion. CNS Neurosci Ther. 2023;29(8):2162–70. 36914967 10.1111/cns.14163 PMC10352867

[17] Wang Z, Fan L. Does stress hyperglycemia in diabetic and non-diabetic acute ischemic stroke patients predict unfavorable outcomes following endovascular treatment? Neurol Sci. 2023;44(5):1695–702. 36652040 10.1007/s10072-023-06625-y

[18] Shen CL, Xia NG, Wang H, Zhang WL. Association of stress hyperglycemia ratio with acute ischemic stroke outcomes post-thrombolysis. Front Neurol. 2021;12: 785428. 35095730 10.3389/fneur.2021.785428 PMC8793935

[19] Merlino G, Smeralda C, Gigli GL, Lorenzut S, Pez S, Surcinelli A, et al. Stress hyperglycemia is predictive of worse outcome in patients with acute ischemic stroke undergoing intravenous thrombolysis. J Thromb Thrombolysis. 2021;51(3):789–97. 32830310 10.1007/s11239-020-02252-y

[20] Dai Z, Cao H, Wang F, Li L, Guo H, Zhang X, et al. Impacts of stress hyperglycemia ratio on early neurological deterioration and functional outcome after endovascular treatment in patients with acute ischemic stroke. Front Endocrinol (Lausanne). 2023;14:1094353. 36777360 10.3389/fendo.2023.1094353 PMC9910688

[21] Stalikas N, Papazoglou AS, Karagiannidis E, Panteris E, Moysidis D, Daios S, et al. Association of stress induced hyperglycemia with angiographic findings and clinical outcomes in patients with ST-elevation myocardial infarction. Cardiovasc Diabetol. 2022;21(1):140. 35883091 10.1186/s12933-022-01578-6 PMC9327277

[22] Chen G, Li M, Wen X, Wang R, Zhou Y, Xue L, et al. Association between stress hyperglycemia ratio and in-hospital outcomes in elderly patients with acute myocardial infarction. Front Cardiovasc Med. 2021;8: 698725. 34355031 10.3389/fcvm.2021.698725 PMC8329087

[23] Esdaile H, Khan S, Mayet J, Oliver N, Reddy M, Shah ASV. The association between the stress hyperglycaemia ratio and mortality in cardiovascular disease: a meta-analysis and systematic review. Cardiovasc Diabetol. 2024;23(1):412. 39550575 10.1186/s12933-024-02454-1 PMC11568630

[24] Marik PE, Bellomo R. Stress hyperglycemia: an essential survival response! Crit Care Med. 2013;41(6):e93–4. 23685597 10.1097/CCM.0b013e318283d124

[25] Monnier L, Colette C. Glycemic variability: should we and can we prevent it? Diabetes Care. 2008;31(Suppl 2):S150–4. 18227477 10.2337/dc08-s241

[26] Tziomalos K, Dimitriou P, Bouziana SD, Spanou M, Kostaki S, Angelopoulou SM, et al. Stress hyperglycemia and acute ischemic stroke in-hospital outcome. Metabolism. 2017;67:99–105. 28081783 10.1016/j.metabol.2016.11.011

[27] Ferrari F, Moretti A, Villa RF. Hyperglycemia in acute ischemic stroke: physiopathological and therapeutic complexity. Neural Regen Res. 2022;17(2):292–9. 34269190 10.4103/1673-5374.317959 PMC8463990

[28] Ahn J, Baik JW, Kim D, Choi K, Lee S, Park SM, et al. In vivo photoacoustic monitoring of vasoconstriction induced by acute hyperglycemia. Photoacoustics. 2023;30: 100485. 37082618 10.1016/j.pacs.2023.100485 PMC10112177

[29] Syed AU, Reddy GR, Ghosh D, Prada MP, Nystoriak MA, Morotti S, et al. Adenylyl cyclase 5-generated cAMP controls cerebral vascular reactivity during diabetic hyperglycemia. J Clin Invest. 2019;129(8):3140–52. 31162142 10.1172/JCI124705 PMC6668679

[30] Espinoza EM, Røise JJ, Li IC, Das R, Murthy N. Advances in imaging reactive oxygen species. J Nucl Med. 2021;62(4):457–61. 33384322 10.2967/jnumed.120.245415 PMC8049370

[31] van Niekerk G, Davis T, Patterton HG, Engelbrecht AM. How does inflammation-induced hyperglycemia cause mitochondrial dysfunction in immune cells? BioEssays. 2019;41(5): e1800260. 30970156 10.1002/bies.201800260

[32] González P, Lozano P, Ros G, Solano F. Hyperglycemia and oxidative stress: an integral, updated and critical overview of their metabolic interconnections. Int J Mol Sci. 2023;24(11):9352. 37298303 10.3390/ijms24119352 PMC10253853

[33] Langen UH, Ayloo S, Gu C. Development and cell biology of the blood-brain barrier. Annu Rev Cell Dev Biol. 2019;35:591–613. 31299172 10.1146/annurev-cellbio-100617-062608 PMC8934576

[34] Mooradian AD. Diabetes-related perturbations in the integrity of physiologic barriers. J Diabetes Complications. 2023;37(8): 108552. 37356233 10.1016/j.jdiacomp.2023.108552

[35] Sweeney MD, Zhao Z, Montagne A, Nelson AR, Zlokovic BV. Blood-brain barrier: from physiology to disease and back. Physiol Rev. 2019;99(1):21–78. 30280653 10.1152/physrev.00050.2017 PMC6335099

[36] Chen H, Guan B, Chen S, Yang D, Shen J. Peroxynitrite activates NLRP3 inflammasome and contributes to hemorrhagic transformation and poor outcome in ischemic stroke with hyperglycemia. Free Radic Biol Med. 2021;165:171–83. 33515754 10.1016/j.freeradbiomed.2021.01.030

[37] Aggarwal A, Yadav A, Saini N, Sandhir R. S-nitrosoglutathione alleviates hyperglycemia-induced neurobehavioral deficits involving nitro-oxidative stress and aberrant monaminergic system. Nitric Oxide. 2022;122–123:35–44. 35257853 10.1016/j.niox.2022.03.001

[38] Huang YW, Li ZP, Yin XS. Stress hyperglycemia and risk of adverse outcomes in patients with acute ischemic stroke: a systematic review and dose-response meta-analysis of cohort studies. Front Neurol. 2023;14:1219863. 38073650 10.3389/fneur.2023.1219863 PMC10701542

[39] Powers WJ, Rabinstein AA, Ackerson T, Adeoye OM, Bambakidis NC, Becker K, et al. 2018 Guidelines for the early management of patients with acute ischemic stroke: a guideline for healthcare professionals from the American Heart Association/American Stroke Association. Stroke. 2018;49(3):e46–110. 29367334 10.1161/STR.0000000000000158

[40] Classification and Diagnosis of Diabetes. Standards of medical care in diabetes-2020. Diabetes Care. 2020;43(Suppl 1):S14-s31. 31862745 10.2337/dc20-S002

[41] Targets G. Standards of medical care in diabetes-2020. Diabetes Care. 2020;43(Suppl 1):S66-s76. 31862749 10.2337/dc20-S006

