# Supplementary material for: Stress hyperglycemia ratio as an important predictive indicator for severe disturbance of consciousness and all-cause mortality in critically ill patients with cerebral infarction: a retrospective study using the MIMIC-IV database
Source: Eur J Med Res. 2025 Jan 27;30:53. doi: 10.1186/s40001-025-02309-9 (PMC11771033; doi:10.1186/s40001-025-02309-9)
Supplement: Supplementary file 1 — Supplementary Material 1. Clinical Advances in the Association Between SHR and Stroke: A Five-Year Review [file 40001_2025_2309_MOESM1_ESM.docx]

**Supplementary material 2**：Clinical Advances in the Association Between SHR and Stroke: A Five-Year Review

| No. | First author | Publication Years | SHR Calculation Method | Study Type | Study Center | Patient Cohort | Overall Sample Size | Clinical Outcome | Outcome Sample Size | Key Findings |
| --- | --- | --- | --- | --- | --- | --- | --- | --- | --- | --- |
| 1 | Guojuan Chen[1] | 2025 | Blood glucose (mmol/L)/ by HbA1c (%). | Retrospective cohort study | The Third China National Stroke Registry | Acute ischemic stroke or transient ischemic attack | 7186 | Functional disability Recurrent occurred Combined vascular events  All-cause mortality | 893 404 420 82 | SHR was linked to functional disability, mediated by stroke severity, but not to recurrent ischemic stroke, transient ischemic attack, combined vascular events, or all-cause mortality. |
| 2 | Yaxin Zhang[2] | 2024 | Blood glucose (mg/dL)) / (28.7 × HbA1c (%) − 46.7) | Retrospective cohort study | MIMIC-IV database (United States) | Acute ischemic stroke | 2029 | 30-day mortality 90-day mortality Hospital mortality | 404 522 309 | SHR is associated with increased 30-day and 90-day all-cause mortality among Acute ischemic stroke patients. |
| 3 | Shiyan Xiao[3] | 2024 | Blood glucose (mmol/L)/ by HbA1c (%) | Retrospective cohort study | The First Affiliated Hospital of Shenzhen University (China) Shenzhen Second People’s Hospital (China) | Acute ischemic stroke | 1255 | 90-day poor functional outcomes | 299 | SHR is associated with increased 90-day all-cause mortality among Acute ischemic stroke patients |
| 4 | Zhouzhou Peng[4] | 2024 | Blood glucose (mmol/L)/ by HbA1c (%) | Prospective cohort study | Cohort study using data from a nationwide prospective registry (China) | Acute basilar artery occlusion stroke treated with endovascular therapy | 250 | 90-day favorable outcome  1-year favorable outcome | 101 234 | SHR was associated with a lower likelihood of favorable outcome at 90 days and 1 year after endovascular therapy in Acute basilar artery patients. |
| 5 | Chunyang Pang[5] | 2024 | Blood glucose (mmol/L)/ by HbA1c (%) | Retrospective cohort study | The First Affiliated Hospital of Wenzhou Medical University (China) | Acute ischemic stroke | 887 | Poor prognosis at 1 year | 157 | Acute post-stroke SHR was independently associated with a poor 1-year prognosis. |
| 6 | Haowei Pan[6] | 2024 | Fasting blood glucose(mmol/L)/ by HbA1c (%) | Retrospective cohort study | MIMIC-IV database (United States) | Severe ischemic stroke | 1376 | 30-day mortality 90-day mortality 1-year mortality | 210 260 322 | SHR may help identify patients at high risk of death after ischemic stroke. |
| 7 | Sarawut Krongsut[7] | 2024 | SHR1：fasting blood glucose (mmol/L)/HbA1c(%); SHR2：admission blood glucose (mmol/L)/HbA1c(%);  SHR3：fasting blood glucose (mmol/L)/(1.59 × HbA1c(%)−2.59;  SHR4：admission blood glucose (mmol/L/(1.59 × HbA1c(%)−2.59 | Prospective cohort study | Saraburi Hospital (Thailand ) | Acute ischemic stroke treated with intravenous rt-PA | 345 | In-hospital mortality Malignant cerebral edema Symptomatic intracerebral hemorrhage 3-month mortality 3-month poor functional outcome | 65 52 42 83 138 | SHR1 is independently associated with fatal outcomes in acute ischemic strok patients treated with rt-PA. |
| 8 | Zhengyang Wang[8] | 2023 | Fasting blood glucose (mmol/L)/HbA1c(%) | Prospective cohort study | Jiangsu Taizhou People's Hospital (China) | Acute ischemic stroke achieved complete recanalization | 209 | Futile recanalization 3-month all-cause mortality | 75 44 | In Acute ischemic stroke patients without diabetes who underwent endovascular treatment, severe SHR independently increased the likelihood of futile recanalization and 3-month all-cause mortality. In contrast, diabetic patients were not affected by SHR. |
| 9 | Yi Sun[9] | 2023 | Random blood glucose (mmol/L)/HbA1c(%) Fasting blood glucose (mmol/L)/HbA1c(%) | Prospective cohort study | The First Affiliated Hospital of Wannan Medical College (China) | Anterior circulation large vessel occlusive stroke underwent mechanical thrombectomy | 423 | 90-day Poor outcomes  Hemorrhagic transformation | 192 111 | SHR is strongly associated with poor 90-day prognosis in mechanical thrombectomy patients and an increased risk of hemorrhagic transformation. |
| 10 | Zhouzhou Peng[10] | 2023 | Blood glucose (mmol/L)/ by HbA1c (%) | Randomized controlled trial | 55 comprehensive stroke centers in China | Acute large vessel occlusion stroke | 542 | 90-day did not achieve excellent outcome 90-day mortality Symptomatic intracranial hemorrhage  Any intracranial hemorrhage | 286 85 39 165 | SHR was associated with decreased odds of achieving a favorable functional outcome at 90 days in patients with acute large vessel occlusion stroke. |
| 11 | Hongbing Liu[11] | 2023 | Blood glucose (mmol/L)/ by HbA1c (%) | Prospective cohort study | The First Affiliated Hospital of Zhengzhou University (China) | Single subcortical infarct | 1049 | Early neurological deterioration 3-month mortality 3-month poor outcome | 206 23 195 | SHR is independently associated with early neurological deterioration and poor outcomes in patients with single subcortical infarct. |
| 12 | Jiahuan Guo[12] | 2023 | Fasting blood glucose (mmol/L)/HbA1c(%) | Retrospective cohort study | Beijing Tiantan Hospital (China) | Acute ischemic stroke or transient ischemic attack | 687 | 90-day poor functional outcomes | 154 | SHR is independently associated with more severe stroke and increased risk of poor functional outcomes in young adults with ischemic stroke or transient ischemic attack. |
| 13 | Zheng Dai[13] | 2023 | Fasting blood glucose (mmol/L)/HbA1c(%) | Retrospective cohort study | Wuxi People’s Hospital of Nanjing Medical University (China)  Nanjing First Hospital, Nanjing Medical University (China) | Acute stroke patients endovascular thrombectomy | 559 | Early neurological deterioration Symptomatic intracranial hemorrhage Mortality within 90 days Favorable outcome | 74 69 81 284 | SHR may be linked to early neurological deterioration and a decreased likelihood of favorable outcomes after endovascular thrombectomy in acute ischemic stroke patients. |
| 14 | J.N. Ngiam[14] | 2022 | Fasting blood glucose (mmol/L)/HbA1c(%) | Retrospective cohort study | A tertiary institution of the National University Health System(Singapore) | Acute ischemic stroke treated with intravenous thrombolysis | 666 | 3-months poor functional outcomes | 305 | SHR appears to be an important predictor of functional outcomes in acute ischemic strok patients undergoing intravenous thrombolysis, with higher SHR independently associated with poor outcomes. |
| 15 | Donghua Mi[15] | 2022 | Fasting blood glucose (mmol/L)/HbA1c(%) | Retrospective cohort study | Chinese Stroke Center Alliance database | Acute ischemic stroke | 168381 | In-hospital death | 882 | SHR may serve as an additional parameter for prognosis in diabetic patients after acute ischemic stroke. |
| 16 | Xin Liu[16] | 2022 | Fasting blood glucose (mmol/L)/HbA1c(%) | Retrospective cohort study | The RESCUE-RE database (China) | Acute stroke with endovascular thrombectomy | 592 | Early neurological deterioration Symptomatic intracranial hemorrhage 90-day mortality Favorable outcome | 74 69 81 284 | SHR is correlated with poor neurological outcomes at 3 months in large artery occlusion acute ischemic stroke patients receiving endovascular therapy. |
| 17 | Guangshuo Li[17] | 2022 | Admission blood glucose (mmol/L)/HbA1c(%); | Retrospective cohort study | Beijing Tiantan Hospital (China) | Acute ischemic stroke treated with intravenous thrombolysis | 294 | Favorable functional outcome at discharge | 175 | SHR was associated with functional outcomes in acute ischemic stroke patients receiving intravenous thrombolysis. |
| 18 | Zhong‑ming Cai[18] | 2022 | Fasting blood glucose (mmol/L)/HbA1c(%) | Prospective cohort study | Five major medical institutions in Wenzhou (China) | Acute ischemic stroke | 971 | 3-months mortality 12-months mortality | 35 85 | SHR was associated with an increased risk of adverse outcomes in acute ischemic stroke. |
| 19 | Chengxiang Yuan[19] | 2021 | Blood glucose (mmol/L)/ by HbA1c (%) | Retrospective cohort study | The First Affiliated Hospital of Wenzhou Medical University (China) | Acute ischemic stroke | 572 | Hemorrhagic transformation | 287 | SHR is significantly associated with an increased risk of hemorrhagic transformation in acute ischemic stroke patients. |
| 20 | Gregory Roberts[20] | 2021 | Blood glucose/(1.59 × HbA1c(%)−2.59 | Retrospective cohort study | Flinders Medical Centre, Bedford Park ( Australia) | ischemic stroke | 300 | Poor outcome at discharge | 81 | SHR provided the best prognostic insight at admission to assess the relationship between stress-induced hyperglycemia and ischemic stroke outcomes. |
| 21 | Giovanni Merlino[21] | 2021 | Fasting blood glucose (mmol/L)/HbA1c(%) | Retrospective cohort study | Udine University Hospital (Italy) | Acute ischemic stroke treated with intravenous thrombolysis | 414 | 3-months poor outcome 3-months mortality Symptomatic intracranial hemorrhage | 155 69 31 | SHR is associated with worse outcome in acute ischemic strok patients undergoing intravenous thrombolysis |

**References**

1. Chen G, Xia X, Zhang Y, Zhang X, Li J, Meng X, et al. Associations between stress hyperglycemia and outcomes in patients with ischemic stroke and TIA: the data comes from the Third China National Stroke Registry (CNSR-III). Metab Brain Dis. 2025;40(1):82.

2. Zhang Y, Yin X, Liu T, Ji W, Wang G. Association between the stress hyperglycemia ratio and mortality in patients with acute ischemic stroke. Sci Rep. 2024;14(1):20962.

3. Xiao S, Gao M, Hu S, Cao S, Teng L, Xie X. Association between stress hyperglycemia ratio and functional outcomes in patients with acute ischemic stroke. BMC Neurol. 2024;24(1):288.

4. Peng Z, Tian Y, Hu J, Yang J, Li L, Huang J, et al. The impact of stress hyperglycemia ratio on short-term and long-term outcomes for acute basilar artery occlusion underwent endovascular treatment. BMC Neurol. 2024;24(1):24.

5. Pang C, Chen Y, Chen Y, Lin E, Pan X, Xu Y, et al. What is the impact of acute endocrine and metabolic alterations on long-term ischemic stroke prognosis: a prospective study. BMC Geriatr. 2024;24(1):859.

6. Pan H, Xiong Y, Huang Y, Zhao J, Wan H. Association between stress hyperglycemia ratio with short-term and long-term mortality in critically ill patients with ischemic stroke. Acta Diabetol. 2024;61(7):859-68.

7. Krongsut S, Kaewkrasaesin C. Performance comparison of stress hyperglycemia ratio for predicting fatal outcomes in patients with thrombolyzed acute ischemic stroke. PLoS One. 2024;19(1):e0297809.

8. Wang Z, Fan L. Does stress hyperglycemia in diabetic and non-diabetic acute ischemic stroke patients predict unfavorable outcomes following endovascular treatment? Neurol Sci. 2023;44(5):1695-702.

9. Sun Y, Guo Y, Ji Y, Wu K, Wang H, Yuan L, et al. New stress-induced hyperglycaemia markers predict prognosis in patients after mechanical thrombectomy. BMC Neurol. 2023;23(1):132.

10. Peng Z, Song J, Li L, Guo C, Yang J, Kong W, et al. Association between stress hyperglycemia and outcomes in patients with acute ischemic stroke due to large vessel occlusion. CNS Neurosci Ther. 2023;29(8):2162-70.

11. Liu H, Yao Y, Zhang K, Zong C, Yang H, Li S, et al. Stress hyperglycemia predicts early neurological deterioration and poor outcomes in patients with single subcortical infarct. Diabetes Res Clin Pract. 2023;200:110689.

12. Guo J, Jia J, Zhang J, Liu X, Li G, Zhao X, et al. Prognostic Value of Stress Hyperglycaemia Ratio in Young Patients with Ischaemic Stroke or Transient Ischaemic Attack. Cerebrovasc Dis. 2023;52(5):526-31.

13. Dai Z, Cao H, Wang F, Li L, Guo H, Zhang X, et al. Impacts of stress hyperglycemia ratio on early neurological deterioration and functional outcome after endovascular treatment in patients with acute ischemic stroke. Front Endocrinol (Lausanne). 2023;14:1094353.

14. Ngiam JN, Cheong CWS, Leow AST, Wei YT, Thet JKX, Lee IYS, et al. Stress hyperglycaemia is associated with poor functional outcomes in patients with acute ischaemic stroke after intravenous thrombolysis. Qjm. 2022;115(1):7-11.

15. Mi D, Li Z, Gu H, Jiang Y, Zhao X, Wang Y, et al. Stress hyperglycemia is associated with in-hospital mortality in patients with diabetes and acute ischemic stroke. CNS Neurosci Ther. 2022;28(3):372-81.

16. Liu X, Nie XM, Pu YH, Yan HY, Pan YS, Liu LP. [The association between stress hyperglycemia ratio and outcome of patients with acute ischemic stroke undergoing endovascular treatment]. Zhonghua Yi Xue Za Zhi. 2022;102(27):2096-102.

17. Li G, Wang C, Wang S, Hao Y, Xiong Y, Zhao X. Clinical Significance of Stress Hyperglycemic Ratio and Glycemic Gap in Ischemic Stroke Patients Treated with Intravenous Thrombolysis. Clin Interv Aging. 2022;17:1841-9.

18. Cai ZM, Zhang MM, Feng RQ, Zhou XD, Chen HM, Liu ZP, et al. Fasting blood glucose-to-glycated hemoglobin ratio and all-cause mortality among Chinese in-hospital patients with acute stroke: a 12-month follow-up study. BMC Geriatr. 2022;22(1):508.

19. Yuan C, Chen S, Ruan Y, Liu Y, Cheng H, Zeng Y, et al. The Stress Hyperglycemia Ratio is Associated with Hemorrhagic Transformation in Patients with Acute Ischemic Stroke. Clin Interv Aging. 2021;16:431-42.

20. Roberts G, Sires J, Chen A, Thynne T, Sullivan C, Quinn S, et al. A comparison of the stress hyperglycemia ratio, glycemic gap, and glucose to assess the impact of stress-induced hyperglycemia on ischemic stroke outcome. J Diabetes. 2021;13(12):1034-42.

21. Merlino G, Smeralda C, Gigli GL, Lorenzut S, Pez S, Surcinelli A, et al. Stress hyperglycemia is predictive of worse outcome in patients with acute ischemic stroke undergoing intravenous thrombolysis. J Thromb Thrombolysis. 2021;51(3):789-97.
